# Characterization of the Immune Response Induced by a Commercially Available Inactivated Bluetongue Virus Serotype 1 Vaccine in Sheep

**DOI:** 10.1100/2012/147158

**Published:** 2012-04-24

**Authors:** Ana Cristina Pérez de Diego, Pedro José Sánchez-Cordón, Ana Isabel de las Heras, José Manuel Sánchez-Vizcaíno

**Affiliations:** ^1^VISAVET Health Surveillance Centre and Animal Health Department, Veterinary Faculty, Complutense University of Madrid, Avenida Puerta de Hierro s/n, 28040 Madrid, Spain; ^2^Department of Comparative Pathology, Veterinary Faculty, University of Córdoba, Animal Health Building, Rabanales Campus, 14014 Córdoba, Spain

## Abstract

The protective immune response generated by a commercial monovalent inactivated vaccine against bluetongue virus serotype 1 (BTV1) was studied. Five sheep were vaccinated, boost-vaccinated, and then challenged against BTV1 ALG/2006. RT-PCR did not detect viremia at any time during the experiment. Except a temperature increase observed after the initial and boost vaccinations, no clinical signs or lesions were observed. A specific and protective antibody response checked by ELISA was induced after vaccination and boost vaccination. This specific antibody response was associated with a significant increase in B lymphocytes confirmed by flow cytometry, while significant increases were not observed in T lymphocyte subpopulations (CD4^+^, CD8^+^, and WC1^+^), CD25^+^ regulatory cells, or CD14^+^ monocytes. After challenge with BTV1, the antibody response was much higher than during the boost vaccination period, and it was associated with a significant increase in B lymphocytes, CD14^+^ monocytes, CD25^+^ regulatory cells, and CD8^+^ cytotoxic T lymphocytes.

## 1. Introduction

Bluetongue virus (BTV), a member of the *Orbivirus *genus in the Reoviridae family [1], shows considerable genetic and antigenic variability, with at least 25 different serotypes characterized to date [2, 3]. These serotypes do not confer cross-protective immunity, which means that specific vaccines must be developed for each serotype [4].

Vaccination has proven very effective in BTV control and eradication strategies [5, 6]. A wide range of vaccines, based on either inactivated or modified live virus, are available against different BTV serotypes [7]. Inactivated vaccines are considered safer than vaccines based on modified live virus because they do not allow the possibility of viral replication. Therefore, they are useful for avoiding virus circulation among susceptible species [8, 9]. Indeed, inactivated vaccines have already been used successfully in field trials, and they are the vaccines most recommended by EU authorities [8, 10]. 

Several inactivated BTV vaccines have been shown to confer protection mainly by inducing production of neutralizing antibodies [11–14]. These antibodies protect primarily against homologous serotypes, and they appear ineffective at cross-protecting against heterologous serotypes [15, 16]. Some studies reveal that cell-mediated immunity could play an important role [17] when vaccinating with individual antigens [18], or concretely just in some sheep [19]. Also, inactivated vaccines have been shown to protect against BTV in the absence of neutralizing antibodies [4, 20], but it is well known that the main effective response against BT is capable to generate neutralizing antibodies [11]. 

Evaluating the cell-mediated immunity in animals vaccinated against BTV could provide valuable information for assessing and improving the efficacy of BTV vaccines. To that end, the present study aimed to examine, in vivo, the cellular and humoral immune response generated by a commercial monovalent, inactivated vaccine against BTV serotype 1 in Merino sheep.

## 2. Materials and Methods

All procedures were carried out in accordance with the Code of Practice for Housing and Care of Animals Used in Scientific Procedures, approved by the European Economic Community in 1986 (86/609/EEC) and amended by European Commission Directive 2003/65/EC. The procedures were also approved by the Animal Experimental Committee of Complutense University of Madrid.

### 2.1. Animals

Five female Merino sheep of 9-10 months old and negative for antigens or antibodies against BTV were housed in Biosafety Level 3 facilities (VISAVET, Complutense University of Madrid).

### 2.2. Vaccine

A commercial inactivated vaccine against BTV serotype 1 was used (Zulvac1, Fort Dodge Veterinaria SA). The active component per dose (2 mL) was BTV-1/ALG2006/01 E1 ≥ 10^6.4^ TCID_50_. This vaccine contains adjuvants such as *Quillaia* bark, with the primary adjuvant being hydrated aluminum hydroxide.

### 2.3. Vaccination, Boost Vaccination, and BTV Challenge

Vaccination was carried out SC on day 0 of the experiment. Booster vaccination was performed by the same route on day 20. In both cases, the vaccine dose was 2 mL as recommended by manufacturer. On day 48, all animals were challenged with 1 mL of BTV1 ALG/2006 at a virus title of 1.9 × 10^6^ TCID_50_ in BHK cells. The challenge inoculum contained 1.9 × 10^6^ TCID_50_ (kindly provided by CISA-INIA), and it was administered intravenously into the jugular vein. On day 68, animals were euthanized. 

### 2.4. Temperature Monitoring, Clinical Survey, and Necropsy

Rectal temperature was measured on day 0 prior to vaccination, as well as on various days until the end of the trial on day 68. On each of these occasions, clinical signs were scored using the system described by Perrin et al. [21].

### 2.5. Sample Collection for Serology and BTV RNA Extraction

Serum samples were collected on day 0 prior to vaccination, as well as on days 3, 14, 16, 20, 21, 23, 26, 35, 42, 48, 51, 53, 54, 57, 58, 61, 62, and 68. Samples were analyzed by a double-recognition ELISA (Ingezim BTV DR 12.BTV.K0, Ingenasa) according to the manufacturer's instructions. Antibody response was measured as optical density.

Samples of EDTA blood were collected on day 0 prior to vaccination, as well as on days 3, 20, 21, 23, 42, 48, 51, 53, 54, 57, 58, 61, 62 and 68. RNA was extracted from the samples using the NucleoSpin RNA II kit (Macherey-Nagel). The presence of BTV RNA was assessed using real-time RT-PCR (RT-qPCR) targeting BTV segment 5. Briefly, this RT-qPCR was able to detect up to 100 RNA copies. The relationship between the Ct and the copy number was linear between 17 and 33 cycles, which correspond to 1 × 10^8^ and 1 × 10^3^ copies, respectively. The RT-qPCR had an efficiency of 96%, and it was associated with an R^2^ of 0.99. The RT-qPCR was able to detect the mRNA in all of the 128 biological samples from sheep, goats, and cattle tested [22]. This could be supposed as a high sensitivity close to 100%. In our study, the negative controls were not template controls, while positive controls where sample from experimentally infected animals, and their Cts were between 20 and 25.

### 2.6. Flow Cytometry Analysis of Peripheral Blood Mononuclear Cells

EDTA blood samples were collected on day 0 prior to vaccination, as well as on days 3, 14, 16, 20, 21, 23, 26, 35, 42, 48, 51, 53, 54, 57, 58, 61, 62, and 68. Flow cytometry using an FACS scan cytometer (Becton Dickinson) was used to detect different populations of peripheral blood mononuclear cells (PBMCs) (Table 1).

### 2.7. Statistical Analysis

Data were analyzed using GraphPad InStat 3.0 and IBM SPSS Statistics 19. Percentages of PBMC subpopulations (CD4^+^, CD8^+^, WC1^+^, CD25^+^, B lymphocytes, and CD14^+^) are reported as mean ± standard deviation (SD). Differences between the percentages of PBMC populations at different times and the values prior to vaccination on day 0 were analyzed by repeated-measures ANOVA with the Huynh-Feldt correction. 

Optical density results from ELISA testing of serum samples are reported as mean ± SD. Differences among mean optical density values after vaccination (days 0–20), after boost vaccination (days 21–48), and after challenge (days 51–68) were analyzed using the Mann-Whitney *U* test for nonparametric distributions. 

For all comparisons, differences for which *P* < 0.05 were considered significant.

## 3. Results

### 3.1. Temperature, Clinical Signs, and Lesions

Hyperthermia (rectal temperature higher than 40°C) was detected in three sheep on day 1 after vaccination and in three sheep on day 21 after boost vaccination. After challenge, however, no increase in temperature was detected in any animal. 

No BTV clinical signs were observed during the experiment. Moreover, necropsy failed to detect gross lesions characteristic of BTV infection. Histopathology confirmed the absence of microscopic lesions characteristic of BTV.

### 3.2. Determination of Antibody Response by ELISA

**Figure 1:**
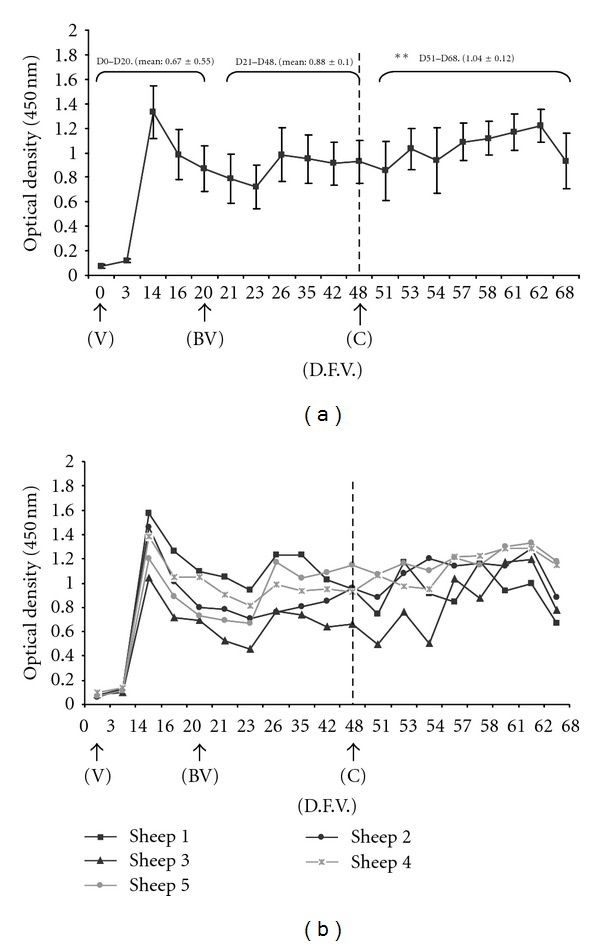
(a) Antibody response in serum samples (mean optical density ± SD) measured by ELISA during the experiment. The threshold below which a response was considered negative was defined as 15% of the positive control optical density. Thus, samples with an optical density > 0.252 were considered positive. The mean value after boost vaccination was significantly different from that after challenge (**P ≤ 0.05; Mann-Whitney *U* test for nonparametric distributions). (b) Individual measurements of antibody response. Abbreviations: V, day of first vaccination; DFV, day after first vaccination; BV, day of boost vaccination; C, day of challenge.

A specific antibody response against BTV was detected in all vaccinated sheep from day 14 through the end of the experiment (Figures 1(a) and 1(b)). Antibody levels peaked on day 14, decreasing moderately thereafter. Antibody levels began to rise again from day 26, and the levels remained relatively constant until day 51. From that point until day 62, the levels increased again, showing a slight decrease only in the final stage of the study.

Mean values of antibody response were compared for the periods after vaccination (days 0–20), after boost vaccination (days 21–48), and after challenge (days 51–68). No significant differences were observed between antibody levels after vaccination (0.67 ± 0.55) and after boost vaccination (0.88 ± 0.1). After challenge, however, the antibody level increased significantly to 1.04 ± 0.12 (Figure 1(a)).

### 3.3. BTV RNA Detection by RT-PCR

No viral genome was detected in the animals at any time in the study. 

### 3.4. Analysis of PBMC Populations

#### 3.4.1. CD4^+^

**Figure 2:**
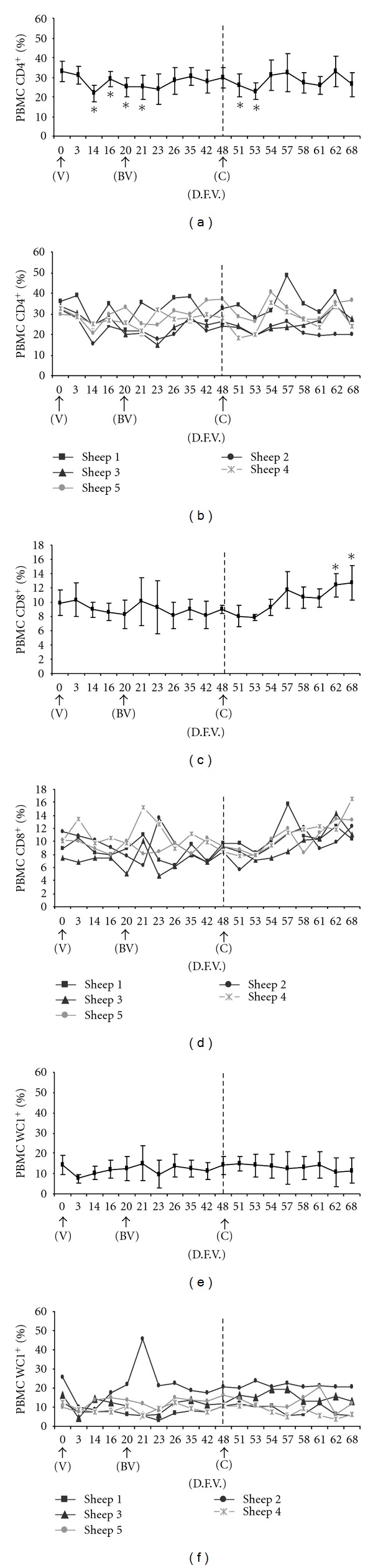
Mean percentages (± SD) and individual percentages of PBMC populations labeled by antibodies against CD4*^+^* (a, b), CD8*^+^* (c, d), and WC1*^+^* (e, f) throughout the experiment. * is statistically significant difference (*P *≤ 0.05) from prevaccination value on day 0, based on ANOVA with the Huynh-Feldt correction. Abbreviations: V, day of first vaccination; DFV, day after first vaccination; BV, day of boost vaccination; C, day of challenge.

CD4*^+^* T lymphocytes represented 32.87% of PBMC before vaccination. Between days 14 and 21, the proportion of CD4*^+^* cells significantly declined, with the minimum value of 21.7% occurring on day 14 (Figures 2(a) and 2(b)). Subsequently, the proportion returned to its prevaccination level. After challenge, the level decreased significantly again between days 51 and 53, reaching 22.7% on day 53. The level then returned to prevaccination values for the rest of the trial.

#### 3.4.2. CD8^+^

The mean percentage of CD8*^+^* T lymphocytes prior to vaccination (day 0) was 9.89% (Figures 2(c) and 2(d)). This percentage did not vary significantly after vaccination or boost vaccination. It increased from day 57, nine days after challenge, and peaked between days 62 and 68.

#### 3.4.3. WC1^+^

The percentage of *γδ* T lymphocytes labeled by the anti-WC1*^+^* antibody did not vary significantly from the prevaccination value of 14.28% (Figures 2(e) and 2(f)).

#### 3.4.4. CD25^+^

**Figure 3:**
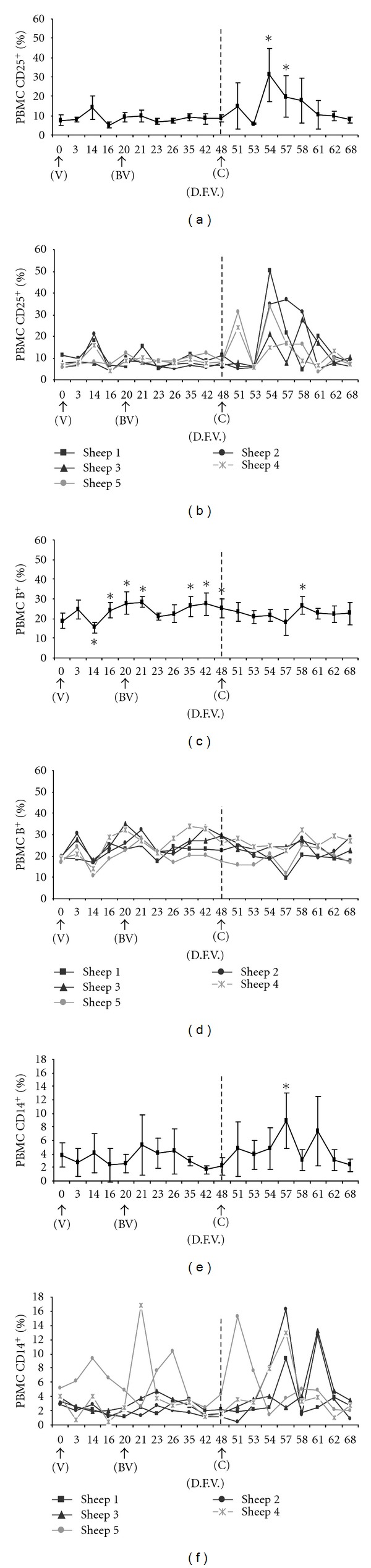
Mean percentages (± SD) and individual percentages of PBMC populations labeled by antibodies against CD25*^+^* (a, b), B*^+^* cells (c, d), and CD14*^+^* (e, f) throughout the experiment. *is statistically significant difference (*P *≤ 0.05) from the mean pre-vaccination value on day 0, based on ANOVA with the Huynh-Feldt correction. Abbreviations: V, day of first vaccination; DFV, day after first vaccination; BV, day of boost vaccination; C, day of challenge.

The percentage of CD25*^+^* cells increased slightly, but not significantly, from a pre-vaccination value of 7.44% to 14.18% on day 14 (Figure 3(a)). This percentage did not change significantly after boost vaccination. After challenge, however, all five animals showed an increase in the proportion of CD25*^+^* cells. For example, by day 51, the level in sheep 4 and sheep 5 had increased, respectively, to 24.2% and 31.3% (Figure 3(b)). The level increased significantly again between days 54 and 57, peaking on day 54 (31.2%). Subsequently, the level decreased progressively and returned to pre-vaccination values at the end of the experiment.

#### 3.4.5. B Lymphocytes

The percentage of B cells decreased significantly after vaccination, with the lowest level of 15.4% occurring on day 14 (Figures 3(c) and 3(d)). The level significantly increased thereafter between days 16 and 21, and again after boost vaccination between days 35 and 48. After challenge, the percentage varied slightly, showing a significant increase on day 58.

#### 3.4.6. CD14^+^

The percentage of monocytes labeled by the anti-CD14*^+^* antibody did not change significantly from its pre-vaccination value of 3.85% after vaccination or boost vaccination (Figures 3(e) and 3(f)). After challenge, however, the percentage increased significantly, reaching 10.28% on day 57. Subsequently, it decreased until it returned to pre-vaccination values on days 62 and 68. After challenge, all animals showed an increase followed by a marked decrease. This response occurred on different dates for different animals, though in all cases the levels reached similar values.

## 4. Discussion

The inactivated vaccine against BTV serotype 1 (Zulvac1, Fort Dodge Veterinaria SA) induced an effective immune response in all vaccinated sheep. Challenge virus was not detected in blood by RT-PCR even at 20 days after inoculation. Except for a temperature increase observed in most animals after the vaccination and boost vaccination, no clinical signs or lesions characteristic of BTV infection were observed.

The temperature increase observed in most animals after vaccination and boost vaccination may reflect activation/stimulation of the immune system. Similar increases were observed after administration of inactivated vaccines against different serotypes of BTV [23, 24]. However, such a temperature increase was not observed in sheep after vaccination or revaccination with vaccines containing virus-like particles [25]. These vaccines lack genetic material and so are nonreplicative; instead, they contain complexes of structural proteins (VP2, VP3, VP5, and VP7) [26]. Therefore, even though inactivated BTV vaccines do not contain live virus, they seem to have a greater ability to stimulate the immune system from a very early stage due to the presence of potent adjuvants. 

After subcutaneous administration of inactivated vaccines against BTV, the presence of viral RNA in blood should not be detected [11, 27]. Moreover, such vaccines prevent viral replication after challenge with homologous virus [28, 29]. Our results showed the absence of virus in blood after both vaccinations as well as after challenge with BTV serotype 1. These findings suggest that this vaccine may prevent both virus dissemination and disease spread from vaccinated animals. Therefore, in the presence of vector-borne BTV serotype 1, the inactivated vaccine not only prevents virus replication but also effectively induces a protective immune response. The specific protection conferred by these vaccines appears to relate to the key role played by structural protein VP2 in stimulating protective immunity mediated by T and B cells [18, 30]. However, the immunological mechanisms behind the protection observed in vaccinated animals, including the possible role of the cellular immune response, remains unclear. 

At 14 days after vaccination, a specific antibody response against BTV was observed in all vaccinated sheep, and it persisted through the end of the experiment. This antibody response was shown to be the main factor in protecting sheep against BTV serotype 1. Our results are in agreement with previous studies [8, 11], wherein the specific antibodies detected by ELISA after administration of inactivated BTV vaccines in sheep were considered to be neutralizing antibodies with the ability to protect against the virus. Nevertheless, how antibodies against BTV neutralize virus *in vivo* remains unclear, despite attempts to demonstrate antibody-dependent cellular cytotoxicity [17, 31]. 

The potential role of the cellular immune response during BTV infection and after vaccination is not fully understood [4, 18, 20, 32]. In the present study, the specific antibody response detected in sheep after vaccination and boost vaccination against BTV serotype 1 was not associated with either a significant increase in T lymphocyte subpopulations (CD4*^+^*, CD8*^+^*, and WC1*^+^*) or in CD25*^+^* regulatory cells. Moreover, significant changes in the percentage of CD14*^+^* monocytes were not observed. These results are in agreement with previous studies of inactivated vaccines against BTV serotype 1 in sheep [33]. 

The lack of cell-mediated immunity after vaccination and boost vaccination in our experiment could be attributed to different components of the vaccine, in which inactivated virus was mixed with an aluminum-based adjuvant. These adjuvants delay the elimination of antigens after vaccine administration, prolonging the antigenic stimulus. These adjuvants also promote antibody response, even though they have little stimulatory effect on cell-mediated responses [34–36]. This inability to stimulate a strong cell-mediated response, together with the diversity of responses by antigen-presenting cells following exposure to live or killed viruses in vaccines [37, 38], may explain the moderate participation of cell-mediated response after vaccination and boost vaccination, as well as the absence of significant changes in the proportions of these immune cell populations. 

On the other hand, B cells can recognize most antigens without prior processing, and certain antigens can provoke antibody formation in the absence of helper T cells, providing sufficient signal for B cell proliferation and differentiation into antibody-producing plasma cells [39, 40]. This may explain why the significant increase in B lymphocytes observed after vaccination and boost vaccination in the present study was associated with an increase in specific antibodies against BTV serotype 1. 

After challenge, viral genome was not detected in vaccinated sheep, confirming specific protection induced by antibody response. In fact, antibody response after challenge was significantly higher than after boost vaccination, and this increase was associated with a significant increase in CD14*^+^* monocytes, CD25*^+^* cells, B lymphocytes, and CD8*^+^* T lymphocytes. Thus, challenge with BTV serotype 1 significantly increased the levels of CD14*^+^* monocytes and CD25*^+^* regulatory cells. CD14 has been defined as a central molecule in antigen recognition and cellular interactions, where it plays a key role in monocyte-mediated T-cell activation [18, 41]. In addition to dendritic cells [32], monocyte macrophages play an important role in antigen presentation and virus spread during BTV infection [42, 43]. CD25 has been defined as a low-affinity receptor for IL-2 that is expressed mainly on T cells activated by interaction with antigens [44, 45]. Thus, the increase in CD14*^+^* monocytes and CD25*^+^* regulatory cells observed after challenge with BTV serotype 1 may costimulate B cells, allowing their activation, proliferation, and differentiation into antibody-producing plasma cells. 

Finally, after challenge with BTV serotype 1, an increase in CD8*^+^* T lymphocytes was observed, which became significant toward the end of the trial. Similar increases in CD8*^+^* T lymphocytes and the cytokine IL-2 in association with an increase in CD25 expression have been described after challenge of sheep vaccinated against BTV serotype 1 [33]. Such increases have also been observed during experimental infections [31, 32, 46]. Thus, the activation and proliferation of CD8*^+^* T lymphocytes, which play a key role in the Th1 response dominated by cytotoxic T cells, may also be associated with the increase in CD14*^+^* monocytes and CD25*^+^* regulatory cells observed after challenge.

## 5. Conclusions

Collectively, these results demonstrate that a specific and protective antibody response was induced after vaccination and boost vaccination of sheep with an inactivated vaccine against BTV serotype 1 (Zulvac1, Fort Dodge Veterinaria SA). This response was associated with a significant increase in B lymphocytes. However, significant increases were not observed in T lymphocyte subpopulations (CD4*^+^*, CD8*^+^*, and WC1*^+^*), CD25*^+^* regulatory cells, or CD14*^+^* monocytes. After challenge with BTV serotype 1, antibody response significantly increased over the level observed after boost vaccination, and this was associated with significant increases in B lymphocytes, CD14*^+^* monocytes, CD25*^+^* regulatory cells, and CD8*^+^* cytotoxic T lymphocytes.

##  Conflict of Interests

None of the authors has any financial or personal relationships that could inappropriately influence or bias the content of the paper.

## Figures and Tables

**Table 1:** Antibodies used to analyze PBMC populations by flow cytometry.

Primary and secondary antibodies	Specificity	Ig isotype	Amount per tube (*μ*g)	Source	Reference
Anti-sheep B lymphocytes	B lymphocytes	IgM	2	VMRD	BAQ44A
Anti-sheep CD4	T helper lymphocytes	IgG_1_	2	VMRD	17D1
Anti-sheep CD8	Cytotoxic T lymphocytes	IgG_1_	2	VMRD	CACT80C
Anti-sheep WC1	*γδ* subset of T lymphocytes	IgG_1_	2	VMRD	IL-A29
Anti-sheep CD25	IL-2 receptor *α*-chain	IgG_1_	2	VMRD	CACT116A
Anti-sheep CD14	Monocytes	IgG_1_	2	VMRD	CAM36A
FITC-conjugated anti-mouse IgG1 (*γ*1)	Mouse IgG1 (*γ*1)	—	0.4	Invitrogen	P-21129
PE-conjugated anti-mouse IgM	Mouse IgM	—	0.4	Sigma-Aldrich	F-9259
